# Simulation in Neonatal Resuscitation

**DOI:** 10.3389/fped.2020.00059

**Published:** 2020-02-25

**Authors:** Aisling A. Garvey, Eugene M. Dempsey

**Affiliations:** ^1^Department of Paediatrics and Child Health, Neonatal Intensive Care Unit, University College Cork, Cork, Ireland; ^2^INFANT Research Centre, Cork, Ireland

**Keywords:** neonatal resuscitation, simulation, medical education, delivery room, quality performance, chest compressions, teamwork

## Abstract

Approximately 1 in 10 newborns will require basic resuscitation interventions at birth. Some infants progress to require more advanced measures including the provision of positive pressure ventilation, chest compressions, intubation and administration of volume/cardiac medications. Although advanced resuscitation is infrequent, it is crucial that personnel adequately trained in these techniques are available to provide such resuscitative measures. In 2000, Louis Halmalek et al. called for a “New Paradigm in Pediatric Medical Education: Teaching Neonatal Resuscitation in a Simulated Delivery Room Environment.” This was one of the first articles to highlight simulation as a method of teaching newborn resuscitation. The last decades have seen an exponential growth in the area of simulation in newborn care, in particular in newborn resuscitation and stabilization. Simulation is best defined as an instructional strategy “used to replace or amplify real experiences with guided experiences that evoke or replicate substantial aspects of the real world in a fully interactive manner.” Simulation training has now become an important point of how we structure training and deliver improved healthcare to patients. Some of the key aspects of simulation training include feedback, deliberate practice, outcome measurement, retention of skills and curriculum integration. The term “Train to win” is often used in sporting parlance to define how great teams succeed. The major difference between sports teams is that generally their game day comes once a week, whereas in newborn resuscitation every day is potentially “game day.” In this review we aim to summarize the current evidence on the use of simulation based education and training in neonatal resuscitation, with particular emphasis on the evidence supporting its effectiveness. We will also highlight recent advances in the development of simulation based medical education in the context of newborn resuscitation to ensure we “train to win.”

## Introduction

Approximately 1 in 10 newborns will require basic resuscitative interventions at birth. A small proportion (<1%) of infants will proceed to require advanced measures including the provision of positive pressure ventilation, chest compressions, intubation and administration of volume or cardiac medications. This need for advanced resuscitation is often unanticipated prior to delivery (1). Given the relative infrequency of such advanced measures it is unlikely that personnel will be exposed to these on a regular basis. However, it is essential that personnel adequately trained in newborn resuscitation are available when such advanced measures are required. A number of standardized newborn resuscitation training schemes are taught around the world. Simulation training has become a central component of these programs.

Simulation is best defined as an instructional strategy “used to replace or amplify real experiences with guided experiences that evoke or replicate substantial aspects of the real world in a fully interactive manner.” Simulation was first introduced to neonatal resuscitation training in the mid-1990s following its success in the field of aviation for training and assessing pilots (2). Simulation allows for the consolidation of theoretical knowledge and practical skills in a risk-free environment. Key components of effective simulation based medical education include deliberate practice, key measures of outcome assessment and appropriate, timely debriefing. Essentially simulation based healthcare is about practice. William Osler stated “Observe, record, tabulate, communicate. Use your five senses… Learn to see, learn to hear, learn to feel, learn to smell, and know *that by practice alone you can become expert*” (3). This very much holds true today. However, we would contend that it is not “practice that makes perfect,” but that “perfect practice makes perfect.”

Simulation training should ensure that the performance repertoire becomes automatic and thus affords spare attentional capacity to allow one to concentrate upon cognitive tasks, such as perception, judgment, and decision making. The Department of Health in the UK in a 2011 document entitled “A Framework for Technology Enhanced Learning” has as it's first recommendation that “healthcare professionals should learn skills in a simulation environment and using other technologies before undertaking them in supervised clinical practice.”

A number of standardized formal resuscitation training programs exist, the most common being the Newborn Resuscitation Program (NRP). The NRP was first introduced in 1987, and has seen a number of changes over the subsequent editions moving from a mainly didactic teaching session to a now more interactive simulation based approach with debriefing as a central component. As with many interventions, it is often difficult to quantify the clinical effectiveness of simulation based medical intervention in newborn care. However, since its introduction into neonatal resuscitation training, simulation has had a significantly positive impact on neonatal outcomes both in the developed and developing world. In Uganda, simulation based learning has been associated with a reduction in mortality (4) and a systematic review assessing the impact of the Helping Babies Breathe programme showed a significant reduction in stillbirth and first day mortality (5). We have previously highlighted the effectiveness of standardized neonatal resuscitation training programs in reducing mortality in the developing world (6).

This review highlights current evidence in the use of simulation based learning for neonatal resuscitation training and its' effectiveness in the attainment and maintenance of high quality skills along with the development of new interventions and technologies in neonatal resuscitation.

## Why Simulation?

First and foremost simulation based medical education makes sense! Louis Halamek describes simulation and debriefing as essentially practice and coaching (7). Patient safety remains the central aspect of high quality patient care. Appropriately trained personnel should be present at deliveries to ensure the best possible outcome. It seems illogical that one would permit junior trainees to attend newborn resuscitations without first having them exposed to various scenarios in a simulation environment. Neonatal resuscitation is a high-pressure, high-risk situation that can be highly stressful for providers. It is important that junior trainees are provided with appropriate direction, debriefing and guidance before having them embark on their journey into the delivery room. It is equally important that senior doctors continue to ensure they remain competent in the provision of high quality newborn resuscitation and learn new techniques in this setting.

In recent years, the number of neonatal trainees has increased considerably. This increase in numbers partnered with increased governance around maximum length of individual on call times in line with working time directives has resulted in reduced exposure of trainees and seniors to emergency events. Also recent developments in the clinical management of neonates (e.g., non-invasive ventilation for preterm infants and no longer routinely performing endotracheal suctioning of the non-vigorous infants with meconium stained liquor at delivery) have also led to a decrease in opportunities to perform certain procedures. Alternative methods need to be in place to ensure providers remain competent. Structured simulation sessions provide this opportunity.

Simulation is beneficial when learning new skills. With over 50% of all adverse events resulting from medical procedures, the old (1890) Halsted's mantra of “See one, Do one, Teach one” is no longer feasible nor acceptable. Simulation provides the bridge from theoretical to practical learning. It should create a risk free, educational environment where the learner is the priority. Thus, it should eliminate fear of mistakes or patient harm allowing for improved skill development and self-confidence.

Simulation also allows the physician of all experience levels to familiarize themselves with new devices and techniques and changes in management before introducing them into the clinical setting. Medical “inflation” is the concept of the increase in the rate of development of new techniques, procedures or devices aimed (or licensed) to improve patient care. It is often difficult to conduct randomized control trials (RCTs) of new devices in the delivery room. For this reason, other methods of gathering evidence and studying new interventions is required. Simulation provides the ideal research environment as it allows scenarios to be replicated in a systematic and structured manner, without impacting on patients well-being.

Likewise, simulation has a central role to play in maintaining professional competency. Some regulatory bodies require clinicians to demonstrate competency on a regular basis by completing a certain number of procedures. Simulation allows for refresher courses and maintenance of these core skills and this is something that should be incorporated into neonatal care.

Teaching teamwork is critical in newborn resuscitation. Resuscitation is most often an interdisciplinary process. It is essential that each person is fully aware of their role and what is required. This can only be taught primarily in a simulation environment. We will explore this further in a later section. Comparisons are typically made with the aviation industry however, we believe a great deal can also be learned from professional sports teams. Typically, they train almost every day. They simulate offensive and defensive moves. They repeat these and repeat these to perfection. They video-record their performance at training (and during games) and analyse these, both as individuals and as a team. Debriefing and feedback are critical components to enhance performance. These teams essentially “train to win.” Their “game day” comes once a week. On game day they record and analyse their performance with numerous metrics, always seeking to enhance their performance. Translate this analogy to newborn resuscitation and you can see that whilst we have come a long way in the last 20 years, there is significant room to improve (7).

The Neonatal Resuscitation Program (NRP) is now in its 7th edition. There have been significant changes in how the course has been delivered over the last 20 years from primarily a didactic program to now a more practical teaching program. The goal of the NRP is to facilitate the acquisition of cognitive, technical and behavioral skills needed to manage newborns at delivery. Simulation and debriefing are seen as essential components, with a greater emphasis placed on teamwork in a simulation setting. Prior to attending the course participants train on the NRP eSim®, which is a new addition and consists of a screen-based simulator designed by Laerdal Medical. The participant receives automated feedback. During the course they participate in skills stations, integrated skills stations and then have a team based simulation with debriefing afterwards. The debrief remains the critical element of this learning process (8, 9).

## Technical Skills

Each step in the resuscitation algorithm is individually important. If completed promptly and effectively, subsequent steps may not be required. For this reason, it is crucial that each skill is optimized which can be difficult when the need for advanced resuscitative steps are infrequent and unpredictable. Teaching technical skills and assessing competency is a crucial aspect of neonatal stabilization. We will review the individual skill components, addressing some of the innovative ways we may improve these necessary skills: IPPV (intermittent positive pressure ventilation), intubation, chest compressions and the administration of medications.

Deliberate Practice is a process where learners are motivated to improve by completing predefined objectives in a systematic way with immediate feedback to advance skills and knowledge (10). Simulation creates a low risk environment where these skills can be observed and direct feedback obtained.

### Provision of IPPV

Providing effective positive pressure ventilation is the cornerstone of neonatal resuscitation with ~5% of term newborns requiring some form of respiratory support. This percentage is significantly higher in preterm infants. Although utilized more frequently than other resuscitation skills, effective PPV is more challenging than perceived. In infants with continued respiratory depression at delivery, two-thirds are thought to be due to ineffective PPV (11). Furthermore, studies have shown that preterm infants in particular are exposed to a wide variety of pressures and tidal volumes in the delivery room, putting the already vulnerable preterm lung and brain at an even greater risk (12, 13).

Optimizing PPV depends on minimizing mask leak, obtaining adequate tidal volumes and inflating pressures and maintaining a consistent rate. Current NRP guidelines state that infants should receive 30 s of effective IPPV before proceeding to the next resuscitative steps (14). Despite this, Skare et al. found that in over 50% of resuscitations PPV was interrupted (15). These pauses in ventilation were more frequent when the resuscitation was unexpected as providers were more stressed and tended to lose focus. Corrective ventilation steps (e.g., MRSOPA) are important to ensure adequate ventilation occurs but can take some time to perform which can delay the initiation of subsequent resuscitative steps (16), highlighting the importance of improved education and vigilance.

Simple demonstration with feedback compared to written instruction alone has been shown to reduce mask leak by up-to 24% (17). When nurses received real-time feedback on chest rise, positioning of mask and ventilation rate, markers of effective ventilation, such as peak inspiratory pressure (PIP), tidal volume and mask leak significantly improved (18). Visual cues have been shown to be important, especially for novice trainees. Mumma et al. found that when SpO_2_ readings were not available, junior respiratory therapists ventilated at a faster rate compared with more experienced staff who placed a greater emphasis on other clinical signs (19).

Familiarity with equipment and its manipulation/ troubleshooting in real-time is a very important component (20, 21). Simulation scenarios provide the ideal resource for achieving this important skill. Understanding the device and its potential adverse effects or limitations is critical. Mathai et al. compared set up times and effectiveness of the T piece and the self-inflating bag, and found that whilst the Neopuff took longer to set up, it was more effective in delivering adequate pressures (22, 23).

Respiratory Function Monitors (RFM) have also been used as a measure of adequate ventilation as they give an estimate of leak and tidal volume and thus can be useful in ascertaining proficiency. Roehr demonstrated how a RFM can provide quantitative and qualitative assessment of the trainee's resuscitation technique, identifying correct mask hold and positioning techniques (24). RFMs have been shown to almost half the incidence of leak during facemask ventilation (25) and when used as part of NRP training, the use of an RFM improved the effectiveness of newborn ventilation (26). They have been used in the clinical environment in one randomized trial which showed that using an RFM was associated with significantly less mask leak, more mask adjustments, and a lower rate of excessive V(T) (27).

Others have evaluated the role of prompts to enhance provision of manual ventilation. Dold et al. evaluated the addition of a musical prompt “Radetzkymarsch” (110 beats per minute) on neonatal CPR and found that in a pilot trial including neonatal trainees that the addition of this musical prompt improved the compliance with the recommended delivery rates of chest compression and manual inflations during neonatal CPR (28). In a similar study the authors evaluated five different auditory prompts and identified that ABBA's “SOS” was the only prompt that improved the rate of chest compressions and inflations provided in a mannequin study. It would appear that in the simulation setting these musical prompts may prove beneficial, but whether or not transferable to the delivery suite is another matter.

### Endotracheal Intubation

Endotracheal intubation is the predominant type of alternate airway used in newborn resuscitation. With recent changes in guidelines regarding management of infants with meconium at delivery and in how preterm infants are managed, opportunities for intubation have reduced significantly. Despite this, intubation remains a core competency for neonatal trainees. Studies have shown that trainees are often unsuccessful at intubation attempts in the delivery room and time taken for each intubation attempt surpasses current recommendations. Furthermore, intubation attempts can have a negative effect on the infant with many deteriorating during attempts (29).

Simulation provides an educational environment where the learner can practice their technique without the time pressure and added stress of a clinically unwell baby. Miller et al. randomized trainees learning to intubate to teaching in the form of pictures and mannequins compared to video and mannequin stations. Those with video were faster at intubation and required less repeated attempts and this difference persisted at 3 months follow up (30). Bensouda et al. examined the effect of trainee stress and performance on neonatal intubation. Trainees were randomized to having 1 vs. 5 healthcare professionals observing their intubation attempts (31). Although there was no difference in time taken to intubate, there was a significant increase in levels of trainee stress which may be helpful in replicating the pressure associated with a real life resuscitation. Whilst this replicates the environment, it must be remembered that most intubation models are of low fidelity and neonatal mannequin airway dimensions may be somewhat different (32). This may be a contributing factor to explain why, in one study, that whilst trainees showed significant improvement in intubation skills immediately post-intervention, this did not translate into improved clinical performance (33).

Additional methods have been evaluated in a simulation environment. We found that the addition of a teaching app (NeoTube) enhances procedural knowledge and performance, and reduces time to successful intubation in a mannequin model (34). Video laryngoscopy in a simulation environment has the potential to improve intubation skills. When compared with direct laryngoscopy, video laryngoscopy improved intubation success rate of trainees and reduced intubation attempt time in a mannequin model (35, 36). Others have evaluated the use of alternative airway devices. The laryngeal mask airway (LMA) is a potential alternative and recent reviews have highlighted the potential of LMA use in neonatal care (37, 38). Their use may become more widespread in neonatal care in the next decade (39).

Simulation does not only apply to trainees but also to experienced physicians who are required to demonstrate competency on an ongoing basis. It is also beneficial in refining and improving skills. Laryngoscopy performed incorrectly can also cause local trauma due to excess pressure applied to the epiglottis and dental arches. One study assessed the pressure exerted by the laryngoscope during intubation and provided real time sound feedback. This resulted in significantly less pressure applied on the second attempt and thus, less trauma (40).

### Chest Compressions

High quality chest compressions are crucial to restoring spontaneous circulation. Studies have shown that prolonged need for chest compressions is associated with decreased survival and poor neurodevelopmental outcomes (41).

Chest compressions are only required in up to 0.3% of deliveries yet they are considered to be a basic skill in newborn resuscitation. To be effective, all components of chest compression provision need to be considered and optimized; hand positioning, rate and depth of compressions as well as their timing with IPPV breaths and allowing for full chest recoil.

Many simulation studies have investigated different rates and techniques of chest compressions which have subsequently informed our current practices. Wyckoff demonstrated that the two-thumb technique was superior to the two-finger technique, achieving greater depth and less variability with each compression in a mannequin study (42). Comparing different rates of compressions, continuous compressions at a rate of 120 beats per minute results in faster performer fatigue and decay in depth of compressions (43, 44). Currently, the International Liaison Committee on Resuscitation (ILCOR) and the NRP recommend that chest compressions are commenced when the heart rate remains below 60 beats per minute despite adequate ventilation, at a ratio of 3 chest compressions to 1 breath. Simulation has also proved beneficial in assessing newer techniques for performing compressions; a new two thumb technique (two thumbs at right angles to the chest with fingers clenched in a fist) has been compared to the traditional two thumb technique (thumbs on sternum with fingers encircling the chest and back for support) and two finger technique. The new two thumb technique has been shown to improve depth of compressions and degree of chest recoil along with reducing provider fatigue (45, 46); outcomes which would be difficult to measure in the true clinical scenario due to the infrequency of the intervention and the emergent nature of the scenario. Simulation training plays an important role in the education of staff as it allows for practice of skills under direct supervision. Performance of chest compression provision improves significantly with direct feedback resulting in better hand positioning, rate of compressions and proportion of complete release (47, 48).

### Adrenaline/Access

Adrenaline is recommended in neonatal resuscitation when the heart rate is <60 beats per minute despite adequate ventilation with 100% fiO_2_ and good quality chest compression provision. Although no studies have examined the optimum timing of adrenaline in the neonatal population, pediatric studies have shown that delay to first adrenaline dose is associated with decreased return of spontaneous circulation and survival (49). Many questions remain to be answered in relation to adrenaline administration (50). Observational studies have shown that in infants requiring adrenaline during resuscitation, only 40% received their first dose within the first 10 min of life, with the median age to first adrenaline dose of 10 min (95% CI 8–14 min) (51). Of the 25 infants who received adrenaline in this study, only 2 infants received adrenaline via the ET tube despite the median time to intubation of 3.8 min compared with 9 min to establish UVC access.

In a porcine model Vali et al. have shown that adrenaline administered via an umbilical catheter resulted in higher plasma epinephrine concentrations and a more rapid return of spontaneous circulation than adrenaline administered via an endotracheal tube (52). In simulation studies, a UVC can be inserted in 105 s (53). However, in real-life cases, this takes longer (51, 54). No RCTs have compared different routes of administration; however, simulation can be used to compare different methods of vascular access insertion. Case reports have shown intraosseous lines to be quick and effective (55–57) and simulation studies have shown that IO access is quicker than UVC insertion in both experienced and non-experienced health care professionals (53, 58). However, at present the NRP prefer UVC placement to peripheral or IO access due to decreased risk of extravasation (14, 59, 60). Until the matter of access in neonatal resuscitation is clearly addressed in further studies, the best course of action is to improve training in UVC placement and create simulation conditions that are as true to real life as possible. Sawyer et al. found that the use of real umbilical cords in simulation provided more realistic scenarios as participants must cut and identify the vessels before placing the UVC while advanced resuscitation is underway (61).

It is an oversimplification to state that the above listed skills are purely technical skills, they all include important cognitive and behavioral aspects. They are conducted by a number of different people who each have various roles, highlighting the critical importance of teaching team performance. Newborn resuscitation is a team activity, teaching non-technical skills is essential and really this aspect has only been explored in the last 10 years or so.

## Teaching Non-Technical Skills

Traditionally, the medical profession had a hierarchical formation with experts at the top working autonomously. The aviation industry showed us that crews that score highly on “behavioral markers,” such as communication, leadership, management, and situational awareness, operate better in critical situations (62–64). *To Err is Human: Building a Safer Health System* first highlighted the importance of teamwork and leadership in healthcare (65) and effective teamwork has since been shown to reduce medical errors (66). So coined the idea of “Making a Team of Experts into an Expert Team” (67). Simulation can be used to develop these non-practical skills (68) and can improve confidence and knowledge (69, 70). Critical aspects of team performance include leadership, teamwork and effective communication.

Several studies have shown the benefit of team training as part of resuscitation courses. The TeamSTEPPS (Team Strategies and Tools to Enhance Performance and Patient Safety) programme resulted in significant improvements in teamwork skills, such as communication, mutual support, and situational monitoring (71). When team training was incorporated as part of the NRP, participants completed the resuscitation in a shorter time, showed better team work including information sharing and maintained better workload management (72, 73). Interestingly, in contrast to other skills, high or low fidelity mannequins do not appear to impact on the development of teamwork or behavioral skills (74), making it a low cost, high yield intervention.

Standardized precise communication seems logical. Yamada et al. assessed the impact of standardized communication techniques. They found a trend toward decreased error rate, time to initiation of PPV, and time to initiation of chest compressions in a neonatal simulation study (75).

Role assignment prior to commencing resuscitation is also key. Sawyer et al. evaluated task-oriented role assignment (TORA), which they defined as the assignment of a specific role, a list of tasks, and a location to stand, to each resuscitation team member. They found that TORA training resulted in better behavioral skills during simulated neonatal resuscitation (76). Greater interaction between the neonatal and obstetrical teams means initiatives, such as this may be very important. For example delayed cord clamping with the ability to provide assisted ventilatory support may be critical to the depressed term infant. This requires multidisciplinary team involvement, with briefing beforehand, role assignment and contingency planning also. Simulation training provides the opportunity to evaluate this approach.

The importance of constructive debriefing cannot be underestimated. Both oral debriefing and use of a video are effective (77). Video debriefing can be beneficial as part of the NRP and has been found to improve teamwork (78). Video assisted feedback in conjunction with oral feedback is hypothetically a superior method of information acquisition and skills retention. It potentially allows for the appropriate consideration of positive and negative aspects of performance. It may allow for trainee self-improvement once they can see themselves perform the task (79). Visual communication in association with verbal communication could potentially allow for a greater understanding of areas for future improvement, in addition to reinforcing trainee progress. Finer et al. found that the videoing of resuscitations led to enhanced debriefing and quality improvement methods to be implemented in the clinical setting (80). A recent review article by Leone provides further insights into the role of video in both training and in clinical care (81).

## High Fidelity vs. Low Fidelity Simulation

The basis of simulation is the replication of real-life scenarios. It can be difficult to replicate exactly the intensity, stress and complexity of an actual neonatal resuscitation however modern technology has allowed for increasingly realistic simulation models and experiences. Many simulation devices are available on the market ranging from 200$ for a basic resuscitation doll suitable for practicing IPPV and CPR, to in excess of $80,000 for high fidelity models which have an audible and palpable pulse and it is possible to perform procedures, such as intubation and cannulation. When addressing fidelity it can relate to the environment (realism of the environment in which simulation takes place), the technology used (hardware and other tools used and any software packages resembles what is used in clinical practice) and finally the psychological fidelity (emotional and behavioral aspects of the real situation). A number of studies have compared high vs. low fidelity with some conflicting findings (61, 82–86). Three of these studies found no difference in performance measures between both modalities (83, 85, 86) and one study identified greater teamwork scores with high fidelity simulation (61). Finan found no difference in stress responses between both groups (84). One consistent finding was that all studies were characterized by a preference for the use of the high fidelity simulator. Although advancements in technology allow for more authentic scenarios, the cost of high fidelity mannequins and resuscitation equipment limits its availability and access. It is important to remember that simple, low cost interventions, such as team work training or the use of real umbilical cords to practice UVC insertion can contribute significantly to realistic training models (61).

Enhanced technological advances have seen the introduction of eye tracking software as a potential aid in both simulation training (87, 88) and in delivery room care (89). Foglia studied 24 providers interacting with a respiratory function monitor during simulated neonatal resuscitation and found that users were willing to wear the glasses and looked at exhaled tidal volume more than any other RFM parameter (88). In a similar study Wagner found that providers use of glasses was acceptable and their use enhanced understanding of providers' gaze and perspective during simulated neonatal airway management (87). One study has assessed visual attention of neonatal team leaders during delivery room resuscitation of preterm infants using eye tracking glasses, documenting the time spent gazing at the infant and at the monitor (89). The incorporation of eye tracking software needs further assessment.

## Retention of Skills

Thus far, we have discussed the benefit of simulation in the acquisition and improvement of skills. However, retention of these skills remains a concern. Studies have shown that a significant decay in skills occurs as early as 2 months after NRP (90–92). This is pertinent as this decay in skills is often unrecognized by the physicians themselves (93). Furthermore, senior clinicians are often tasked with the supervision and teaching of resuscitation skills to junior or inexperienced staff at the bedside (94) which may result in overall performance below acceptable standards.

Booster sessions have been shown to improve retention (95) but are required to be quite frequent to be of benefit (95, 96). Different methods of improving retention have been examined. Frequent, short, targeted sessions may be more feasible while maintaining benefit (95, 97, 98). The most recent ILCOR guidelines have highlighted the necessity to ensure that neonatal task training occur more frequently than every 2 years (99).

Similar to simulation, board games aim to encourage active learning among peers (100–102). Cutumisu et al. used a board game to assess the retention of knowledge and found that knowledge increased by 12% (103). Computer based games have also been developed specifically for neonatal resuscitation. They aim to motivate and encourage learning in an enjoyable and novel manner. The NRP curriculum requires participants to complete an online simulation before attending the practical, hands-on session (14). Bulitko et al. also developed the RETAIN computer game which allows players to navigate different resuscitation scenarios which resulted in improved retention following resuscitation courses (104).

## Future Developments

Carol Dweck first described the concept of a “growth mindset” and how it can influence an individual's motivation and ability to learn (105). A growth mindset implies that intelligence or ability is not something that you are born with, instead it is something that can be developed and improved. Individuals with this mindset thrive on failures and set-backs and instead of admitting defeat, they strive for improvement, pushing themselves beyond their level of comfort and capabilities. Excellence does not necessarily come from experience but from hard work and increased effort (106). Studies have shown that lower levels of growth mindset negatively affects performance in neonatal resuscitation (107). Future studies may address the implications of growth mindset interventions on performance and retention of knowledge and skills.

Virtual reality has the potential to build on this further by facilitation of full immersion in a resuscitation scenario (108). Used in training of emergency skills, no study has looked at its use in neonatal resuscitation. It has the advantage of realistic scenarios involving all senses that is reproducible and without facilitator variation. It would also allow for trainees to practice skills in their own time and reduce the need for facilitators and resources needed for simulation. Self-directed teaching which would necessitate appropriate metric characterization upon which to gauge one's proficiency over time, the concept of proficiency based progression to achieve competency.

Additional cognitive aids may assist, such as the use of smart watches which been shown to be useful in the resuscitation scenarios. They can be used during chest compressions to give feedback on the rate and depth of compressions and thus improve the quality of compressions (109).

Enhancing individual and team performance through simulation will remain a key element of any educational intervention. Eye tracking software and smart watches represent technological advances that may enhance our understanding of how teams function and allow us to create an individual team, rather than a team of individuals. We are now entering an era of enhanced monitoring of newborns in the delivery room. Each patient can generate thousands of data points, opening the door to the application of deep learning and the identification of weak signals not perceptible by human operators in the delivery room. This type of monitoring and our ability to interact with it will need to be studied extensively in the simulation environment before it becomes a reality in the delivery room. On another note we need to strive to ensure that briefing and role assignment, both low cost interventions, form part of every resuscitation scenario.

## Conclusion

Neonatal resuscitation is a high risk, low occurrence (HALO) situation. Even in tertiary centers with highly experienced teams, resuscitations guidelines are not strictly adhered to in over 90% of cases (110). We cannot assume that resuscitation certification and competence are interchangeable as immediately after training courses, skills begin to decay to below a level required to pass. In busy neonatal centers it can be difficult to ensure all staff have equal and sufficient exposure to skills and resuscitation events to maintain a high level of competency yet care providers must demonstrate mastery of the basics even under pressure. Simulation based medical education has changed the way we teach and has been shown to be superior to the traditional approach to clinical teaching (111). Neonatal resuscitation programmes now utilize simulation as a key component of their course content. Simulation allows for regular refreshers, deliberate practice and instant feedback where the learner is priority without the risk of jeopardizing patient safety. As neonatologists, we have a responsibility, not only to our patients but to ourselves and our peers that we are all trained to the highest standard. Moving forward, we must look to our own growth mindset to discover ways to improve our own skills and how we teach and prepare staff for these infrequent but crucial events. A greater focus on team performance and communication will ensure that we “train to win.”

## Author Contributions

All authors contributed to the conception of the work, drafting and editing of the manuscript, and approve of the final draft for submission.

### Conflict of Interest

The authors declare that the research was conducted in the absence of any commercial or financial relationships that could be construed as a potential conflict of interest.
